# Optimizing blue poo: A validated, cost-effective method for measuring whole gut transit time

**DOI:** 10.1016/j.mex.2025.103741

**Published:** 2025-11-26

**Authors:** Cyra Schmandt, Julia Trunz, Claudio Perret, Anneke Hertig-Godeschalk, Zeno Stanga, Jivko Stoyanov

**Affiliations:** a Swiss Paraplegic Research, Nottwil, 6207, Switzerland; b Graduate School for Cellular and Biomedical Sciences, University of Bern, Bern, 3012, Switzerland; c Faculty of Health Sciences and Medicine, University of Lucerne, Lucerne, 6002, Switzerland; d Division of Diabetes, Endocrinology, Nutritional Medicine and Metabolism, University Hospital and University of Bern, Bern, 3010, Switzerland; e Institute of Social and Preventive Medicine, University of Bern, Bern, 3012, Switzerland

**Keywords:** Gastrointestinal tract, Gastrointestinal disease, Validation study, Blue dye method, Digestion

## Abstract

Whole gut transit time (WGTT) provides essential insights into gastrointestinal health, but traditional measurement methods are often expensive or invasive. This study optimizes and validates the "blue dye method," an affordable and minimally invasive approach to WGTT measurement. Using "Hollinger Farbpulver Blau" (containing food colors E131 and E132), dye concentrations ranging from 30 mg to 241 mg were tested across four modes of delivery: capsule with liquid, gummy bear, muffin, and capsule with rice crackers and liquid. Each presented limitations: capsules taken with liquid led to inconsistent transit times, gummy bears caused staining, and muffins were perishable. Measured WGTTs varied between 18 and 29 h depending on the mode of delivery and dye concentration. Optimal protocol was a capsule containing 60 mg of dye taken with two rice crackers and liquid, ensuring accurate detection without practical inconveniences. The standardized and optimized blue dye method provides valid WGTT measurements, making it well suited for large-scale population studies and clinical applications.

Uses a simple blue dye as a marker for gut transit.

Tested several modes of delivery and concentrations to find the most practical option.

Established a standardized protocol for reliable and reproducible measurement.

## Graphical abstract

**Figure:**
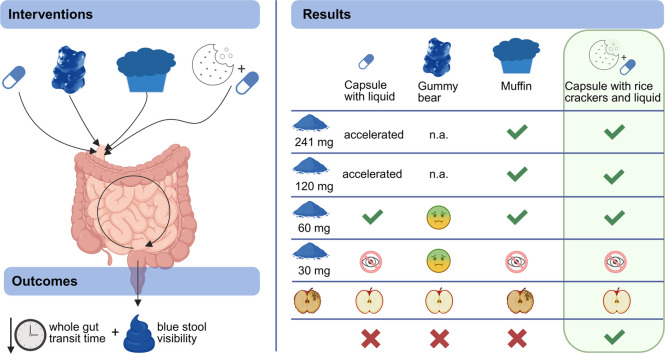

Graphical abstract summarizing the optimized blue dye method developed in this study: It illustrates four modes of delivery tested for administering blue food dye: capsule with liquid, gummy bear, muffin, and capsule with rice crackers and liquid. Furthermore, it shows the four dye concentrations that were tested: 241 mg, 120 mg, 60 mg and 30 mg of colorant. The capsule ingested with liquid showed inconsistent transit times. The gummy bear was unpalatable and caused staining of fingers, teeth, and mouth, reducing user acceptability. The muffin functioned well but was perishable, limiting practicality. The optimal mode of delivery, a capsule ingested with rice crackers and liquid, achieved reliable and accurate measurements of whole gut transit time, without the disadvantages of other modes of delivery. The optimal concentration was 60 mg of colorant, as this represented the lowest dose at which blue stool was reliably observed. Created in BioRender. Schmandt, C. (2026) https://BioRender.com/jgou2z3

## Specifications table

**Table:** 

Subject area	Immunology and Microbiology
More specific subject area	Direct method to measure an indicator of the functionality of the gastrointestinal tract
Name of your method	Blue dye method to measure whole gut transit time
Name and reference of original method	Asnicar F, Leeming ER, Dimidi E, Mazidi M, Franks PW, Al Khatib H, et al. Blue poo: impact of gut transit time on the gut microbiome using a novel marker. Gut. 2021;70(9):1665–74.
Resource availability	Blue dye: https://www.aromen-online.ch/app/easyshop/info?partno=05110HO&seo=Hollinger+Farbpulver+Blau+250*g*&UID=935707–3492A612E9E0D3E3_19303A08A02F. Capsules: KLARE LEERE GEMÜSE KAPSELN (Grösse "1″) von Capsule Connection - BIOVEA SCHWEIZ. Rice crackers: M-Classic · Reiswaffel · Glutenfrei • Migros Online

## Background

The passage of food through the gastrointestinal (GI) tract, from ingestion to excretion, involves complex interactions within diverse physiological environments, notably the acidic stomach and the microbe-rich colon [1]. Various factors, including diet, physical activity, demographics, stress, and medication usage, significantly influence the composition and function of colonic microbiota [1,2]. Whole gut transit time (WGTT), defined as the duration it takes for food to travel through the GI tract, provides a practical measure of digestive function [1,2]. Abnormal transit times, either accelerated or delayed, are associated with GI disorders such as irritable bowel syndrome (IBS) and chronic constipation [1,3,4]. Thus, precise measurement of WGTT is crucial for diagnosing and managing these gastrointestinal conditions [3].

A variety of methods are currently employed to measure gut transit time. Indirect approaches, including the Bristol Stool Scale and fecal water content analysis, assess stool characteristics and predominantly reflect colonic transit time [1,5]. In contrast, direct methods, such as radio-opaque markers, scintigraphy, wireless motility capsules (SmartPills), and 3D-Transit electromagnetic tracking systems, provide comprehensive whole gut transit data but are resource-intensive and require specialized equipment [3,[6], [7], [8]]. Direct methods also demand high levels of participant involvement, as ingested markers are tracked and observed either through X-rays or external detector plates, often requiring extended time in clinical settings.

A simpler and less costly direct method involves consuming identifiable food items, such as sweet corn. However, this method presents practical limitations due to the large quantity of food required [9,10]. A promising alternative, known as the "blue dye method" or "blue poo method", involves the ingestion of edible delivery vehicles containing blue food dye, allowing for easy visual detection of colored stools [11]. A study involving 863 healthy individuals showed that this approach is practical, cost-effective, and minimally invasive [11]. Moreover, it may offer a more informative marker of gut microbiome function than traditional metrics such as stool consistency and frequency. Despite its potential, the blue dye method lacked specificity regarding dye concentrations, making standardized replications challenging [11]. In the original study, the amount of dye-containing paste added to two muffins was reported; however, the exact concentration of the paste was not disclosed. Consequently, the precise quantity of colorant ingested by the participants remains unclear.

Therefore, the current study aimed to optimize and validate the blue dye method for accurately measuring WGTT by identifying the most suitable mode of delivery for dye administration and determining the optimal dye concentration required for reliable stool coloration. In addition, a standardized protocol was established to enable straightforward and consistent replication, making the method suitable for larger-scale population studies.

## Method details

### Setting and study population

This was a prospective, pilot study conducted at the Swiss Paraplegic Research in Nottwil, Switzerland. This pilot study was conducted to validate the blue-dye method for a planned larger project, where this method will be used. The study does not pose any risk to participants and does not require ethics clearance, as it is limited to five healthy volunteers, as defined under the Swiss Human Research Act (HRA art. 2&3, SR 810.30). The volunteers had an average age of 31 years (3 males, 2 females), had no GI symptoms, and maintained their usual diets. Additionally, they were asked not to consume any foods that could potentially color their stool.

### Materials

To identify the most suitable mode of delivery and optimal dye concentration, four delivery methods were evaluated: (1) capsule with liquid, (2) gummy bear, (3) muffin, and (4) capsule with rice crackers and liquid (see Table 1). A blue dye powder (Hollinger Farbpulver Blau, Günter Aroma AG, Beinwil, Switzerland) was used across all modes of delivery. The dye consisted of food colorants E131 (Patent Blue V) and E132 (Indigo Carmine) in a sodium chloride base, with a color concentration of 241 mg/g. The manufacturer’s recommended maximum daily intake corresponded to 1 g of dye powder, equivalent to 241 mg of food colorants. For clarity, all reported values refer to the amount of ingested food colorant rather than the total dye powder used. Four dye concentrations were tested: 241 mg (maximum concentration), 120 mg (50 %), 60 mg (25 %), and 30 mg (12.5 %) of colorant.

**Table 1:** The composition of the four modes of delivery and their concentrations tested in this study.

**Capsule with liquid**	Vegetarian capsules (0.68 ml volume) (Klare leere Gemüse Kapseln Grösse 1, BIOVEA) were filled with 4 different concentrations of the colorant. A single capsule could hold a maximum of 120 mg of colorant; therefore, two capsules were required to achieve the maximum intake of 241 mg. Capsules were ingested with 200 ml of water. The tested intake levels, from highest to lowest colorant dose, were as follows:
	(1) 2 capsules and 200 ml water, colorant ingested: 241 mg
	(2) 1 capsule and 200 ml water, colorant ingested: 120 mg
	(3) 1 capsule and 200 ml water, colorant ingested: 60 mg
	(4) 1 capsule and 200 ml water, colorant ingested: 30 mg
**Gummy bear**	Vegan gummy bears were prepared using agar-agar (Betty Bossi Agar-Agar, Coop, Switzerland). Initially, 4 g agar-agar dissolved in 100 ml water was mixed with 150 ml apple juice (M-Classic Apfelsaft, Migros, Switzerland). Each gummy bear (10 ml) contained 60 mg of colorant.
	A second trial used gummy bears made with 2 g agar-agar in 100 ml water, each containing 30 mg colorant. The tested intake levels, from highest to lowest colorant dose, were as follows:
	(1) 1 gummy bear, colorant ingested: 60 mg
	(2) 1 gummy bear, colorant ingested: 30 mg
**Muffin**	Vegan, gluten-free, lactose-free muffins were prepared following a published recipe (Carine Claudepierre, Vegan Madeleines, The Conscious Plant Kitchen) and freshly baked for each dye concentration. The tested intake levels, from highest to lowest colorant dose, were as follows:
	(1) 1 muffin, colorant ingested: 241 mg
	(2) 1 muffin, colorant ingested: 120 mg
	(3) 1 muffin, colorant ingested: 60 mg
	(4) 1 muffin, colorant ingested: 30 mg
**Capsule with rice crackers and liquid**	Vegetarian capsules (0.68 ml) filled with blue colorant were ingested with 2 store-bought gluten-free rice crackers (M-Classic Vollkornreiswaffeln, Migros, Switzerland) and 100 ml of water. The tested intake levels, from to highest to lowest colorant dose, were as follows:
	(1) 2 capsules with 2 rice crackers and 100 ml water, colorant ingested: 241 mg
	(2) 1 capsule with 2 rice crackers and 100 ml water, colorant ingested: 120 mg
	(3) 1 capsule with 2 rice crackers and 100 ml water, colorant ingested: 60 mg
	(4) 1 capsule with 2 rice crackers and 100 ml water, colorant ingested: 30 mg

### Intervention

Across the cohort, 14 interventions were conducted spanning four modes of delivery; each participant completed a predefined subset with ≥2-day washouts: capsule with liquid (4 concentrations), gummy bear (2 concentrations), muffin (4 concentrations), and capsule with rice crackers and liquid (4 concentrations). Results from each intervention were analyzed immediately and informed subsequent interventions, including adjustments to mode of delivery and concentration.

The intervention began with the capsule with liquid at the highest concentration, which was halved in successive trials as long as the blue color remained visible. The gummy bear was first tested at 30 and 60 mg, but this trial was terminated after two attempts. The muffin and capsule with rice crackers and liquid were tested at the same four concentrations as the capsule with liquid. This design enabled a comparative analysis of the advantages and limitations of each mode of delivery.

Each mode of delivery was administered in the morning following an overnight fast of at least 8 h, during which only water was permitted. Participants ingested the capsule with liquid, gummy bear, and muffin each with approximately 200 ml of water. For the capsule with rice crackers and liquid intervention, participants first consumed two rice crackers, followed by the capsule with approximately 100 ml of water (equivalent to an espresso cup).

Participants recorded the precise ingestion time, the first appearance of blue-colored stool, subsequent occurrences of blue stools, and the first stool without visible blue coloration. A washout period of at least two days was implemented between interventions to prevent interference from residual dye. WGTT, defined as the interval from ingestion of the dye to the first visible appearance of blue-colored stool, and stool coloration visibility were self-reported. Only concentrations and delivery modes that ensured stool visibility in all participants were considered for selection as the optimal dye and concentration. Participants also provided feedback on user-friendliness and reported any potential side effects.

## Method validation

### Visibility and WGTT

To find the best combination of mode of delivery and its concentration the validation process focused on two parameters: stool coloration visibility and WGTT.

All modes of delivery tested produced some degree of stool coloration, typically blue-green and spinach-like-green in appearance. Visibility was categorized as clearly visible, barely visible, or absent, based on participant reports.

Stool color intensity varied according to both dye concentration and mode of delivery.

Higher concentrations (120 – 241 mg) consistently produced strong, complete coloration across all participants, regardless of whether the dye was delivered in a solid medium or in liquid. These high doses were administered initially to ensure clear visual detection of blue-colored stool and typically resulted in multiple visible blue stools, with at least two being very prominent.

Intermediate concentrations (60 mg) produced variable results depending on the mode of delivery. Capsules with liquid yielded fully colored stool, while solid forms resulted in partial coloration, both exhibited strong visibility. The lowest concentration (30 mg) generally produced poor visibility due to insufficient dye content and was tested to determine the minimum effective dose.

A dose of 60 mg of blue dye was identified as the lowest quantity required to ensure reliable stool visibility across all participants, independent of the mode of delivery.

**Fig. 1:**
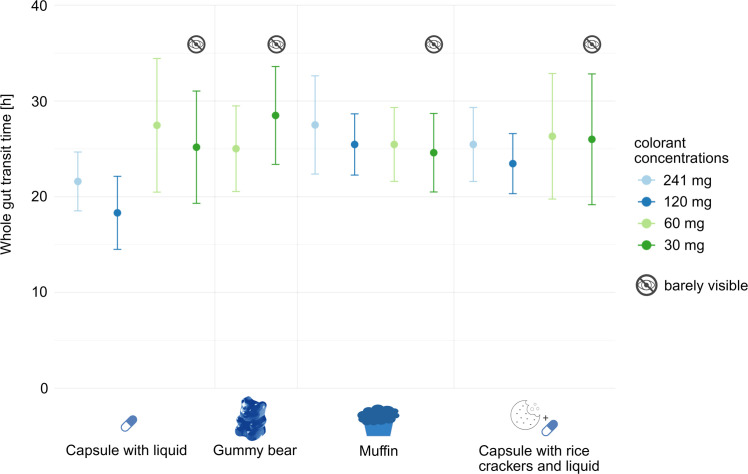
Comparison of mean whole gut transit time (in hours) for the four different modes of dye delivery and four different colorant concentrations. Created in BioRender. Schmandt, C. (2025) https://BioRender.com/bmnjaxt.

WGTT varied depending on the mode of delivery, as shown in Fig. 1. Capsule ingested with liquid had the shortest mean transit time at 23 h 08 min (mean over all concentrations). Co-ingestion with food produced longer transit times: 26 h 45 min for the gummy bear, 25 h 45 min for the muffin, and 25 h 18 min for the capsule with rice crackers and liquid. These values are consistent with previously published data, which report a median WGTT of 28 h 52 min in a cohort of 111 participants using a 3D-Transit electromagnet tracking system [8]. Similarly, Asnicar et al. reported a median WGTT of 28.7 h, with the blue dye ingested on an empty stomach in their protocol [11].

Compared to the muffin (to compare to the original Asnicar study), the capsule with liquid showed a 10 % shorter transit time, indicating its tendency to accelerate gut transit. In contrast, the capsule with rice crackers and liquid had only a modest 2 % shorter transit time relative to the muffin.

WGTT also varied depending on the dye concentration (Fig. 1). The highest concentration (241 mg) yielded a mean WGTT of 24 h 51 min (mean over all modes of delivery), while 120 mg resulted in 22 h 24 min. Notably, these high concentrations display that the capsule with liquid shortens mean transit time compared to other modes of delivery (Fig. 1). When the capsule with liquid data were excluded, the mean WGTT increased by approximately 2 h (241 mg: 26 h 28 min; 120 mg: 24 h 27 min), though this mean was based only on the two remaining modes of delivery. Furthermore, at these high concentrations, transit time was 25 % shorter for the capsule with liquid compared to the muffin, suggesting that co-ingestion with food mitigates the artificially fast transit observed with the capsule taken with liquid alone. Based on these findings, subsequent experiments minimized the amount of water consumed with the capsule and rice crackers to avoid replicating this accelerated transit effect.

At lower concentrations, 60 mg of colorant resulted in a mean WGTT of 26 h 3 min, and 30 mg yielded 25 h 20 min. For these concentrations, the capsule with liquid did not reduce transit time; in fact, at 60 mg, it produced the highest WGTT among all modes of delivery. This may indicate normal gut transit at lower dye concentration, with the dye coloring the food more locally rather than widespread. For the 30 mg concentration, the capsule with liquid showed the lowest mean WGTT; however, at this dose, participants reported barely visible or absent stool coloration, making these values less representative.

WGTT remained relatively stable across all four concentrations for the muffin and the capsule with rice crackers and liquid, whereas the capsule with liquid showed greater variability, as reflected by a higher dispersion (SD 4.0 h vs 1.2 h for the muffin and 1.3 h for the capsule with rice crackers and liquid).

In our study, WGTT values clustered around 24 h, with all participants showing a peak at this time. This suggests that our participants were situated in the faster segment of the WGTT distribution, consistent with Nandhra et al. [8], who observed peaks at approximately 24 and 48 h. Capsule expulsion predominantly occurred in the morning, a pattern also noted in previous studies, highlighting the influence of circadian rhythms on gut motility [8,12].

WGTT appears to be very individual and influenced by multiple factors [[12], [13], [14]]. Daily routines and lifestyle variables, such as the day of the week, sleep patterns, coffee consumption, or simply limited bathroom access during busy workdays likely contribute to individual variation. Additionally, dietary intake, which was not controlled in this study, may have impacted transit times. For instance, a previous study demonstrated that consuming 9 g of dietary fiber over three days significantly reduced WGTT [15].

It is worth noting that the range defined as "normal" WGTT is broad. For example, Lee et al. [16] classified rapid transit as <10 h and delayed transit as greater than 73 h.

### Choice of mode of delivery

Four distinct modes of dye delivery were evaluated in this study, each tested over the range of 30 – 241 mg of colorant. The combination of the capsule with rice crackers and liquid at a color concentration of 60 mg was selected over other modes of delivery, including the capsule with liquid, gummy bear, and muffin, as well as other concentrations (241 mg, 120 mg and 30 mg). A comprehensive overview of the different modes of delivery and concentrations is presented in Table 2.

**Table 2:** Comparison of the four different modes of delivery, including a picture, stool visibility, dye concentrations, and their positive and negative affiliations.

Mode of delivery		Capsule with liquid	Gummy bear	Muffin	Capsule with rice crackers and liquid
Picture					
Visibility at color concentrations (mg)	30	Absent or barely visible	Absent or barely visible	Absent or barely visible	Barely visible
	60	Clearly visible	Clearly visible	Clearly visible	Clearly visible
	120	Clearly visible	–	Clearly visible	Clearly visible
	241	Clearly visible	–	Clearly visible	Clearly visible
Advantages		Simple preparation; suitable for people with allergies/intolerances	Suitable for people with allergies/intolerances	Palatable	Simple preparation; suitable for people with allergies/intolerances
Disadvantages		Inconsistent measurement (visibility and WGTT)	Poor taste; stains hands, mouth, and teeth; lengthy preparation	Short shelf life; lengthy preparation (especially to ensure no contamination with gluten/lactose)	No disadvantages. Consistent across all concentrations.

The capsule with liquid was initially tested as a straightforward method for administering the blue dye powder. This mode of delivery produced very clear stool coloration at all concentrations, except the lowest (30 mg). However, the large variability of mean transit times rendered it unsuitable for the objectives of this experiment. The shortened transit time of the higher concentrations in the capsule with liquid was likely due to liquid-like gastric emptying and rapid passage into the colon [17]. While this effect was not found at lower dye concentrations, the results were too inconsistent to be considered reliable.

To achieve transit times more aligned with other literature, gummy bears were evaluated as a potential mode of delivery. As a solid food with firm texture, long shelf life and nice taste, it seemed like a good option for the dye delivery. However, they were excluded after testing only two concentrations (60 mg and 30 mg) due to poor taste feedback and their tendency to stain the hands, teeth, and mouth blue, even with the lowest amount of dye.

Muffins were then baked to directly compare with the approach used by Asnicar et al. [11]. While they were palatable at lower doses (30 mg, 60 mg, 120 mg), the salty-tasting dye at 241 mg of colorant made them unpalatable and additionally colored the mouth blue. The muffin showed literature-similar WGTT, and stool coloration functioned adequately at doses 60 mg, 120 mg and 241 mg, but their short shelf life and time-intensive preparation rendered them impractical. Although it is relatively easy to bake muffins that are vegan, gluten-free, and lactose-free, there remains a risk of cross-contamination, an issue that can be more reliably mitigated by using a capsule.

To address the mentioned limitations, rice crackers were incorporated into the formulation alongside the capsule with liquid. The capsule was simple to prepare, shelf-stable, and suitable for individuals with allergies. The rice crackers were convenient, commercially available with standardized quality, inexpensive, and gluten-free, making them suitable for a wide range of participants. The hypothesis was that the rice crackers would absorb the blue dye and behave more like solid food as they passed through the GI tract. This combination achieved transit times comparable to the other solid foods consumed. Stool coloration was clearly visible for concentrations from 60 mg to 241 mg. Only the lowest concentration of 30 mg was barely visible.

Overall, 60 mg of color was the lowest concentration that reliably produced visible stool coloration in all participants and modes of delivery. The higher doses were not suitable, in which they rendered the mode of delivery unpalatable or inaccurate. The lowest dose (30 mg of color) was too low, as the blue color was not observed in the stool anymore. Notably, none of the delivery methods resulted in reported side effects.

This paper provides clear and practical guidance for applying the blue dye method in clinical research. It defines optimal modes of delivery and concentrations, while outlining the advantages and limitations of tested approaches to support flexible study design. For dietary intervention studies, the dye can be easily incorporated into simple foods such as muffins, enhancing feasibility and participant compliance. Capsules with rice crackers and liquid emerged as particularly practical formats, being easy to standardize, ship, and use globally without specialized clinical settings. The method also offers a low-cost alternative, suitable for large-scale studies. Although less detailed than advanced technologies like wireless motility capsules, the blue dye method remains a valid, non-invasive, and cost-effective measure of whole gut transit time, balancing scientific rigor with real-world applicability.

## Limitations

This study has several limitations that should be considered when interpreting the results. First, the sample size was small and limited to healthy individuals, which may restrict generalizability to broader or clinical populations, including individuals with GI disorders. Second, although all participants followed the same protocol, self-reported outcomes such as stool color visibility and time of passage may be subject to reporting bias or error. Finally, three data points were missing due to participant unavailability; however, the primary conclusions remain robust, as the selected mode of delivery and concentration consistently yielded visible stool coloration and plausible transit times across the evaluable dataset.

## Ethics statements

This methodological validation study involved no invasive procedures, only the ingestion of food-grade blue dye followed by observation of their own stool, in a cohort of five healthy adult participants. As the data collected from these participants for method validation can be fully and irreversibly anonymized and reported in aggregated form, ethics approval was not required.

## Additional information

Muffin Recipe:

Ingredients used for the recipe (for approx. 5 muffins à 30 g):

• 30 g coconut yoghurt (Migros, V-Love Bio Classic Coconut)
• 30 g oat milk (Migros, V-Love Oat Drink Calcium)
• 75 g sugar (Migros, MClassic Feinkristall Zucker)
• 30 g canola oil (Florin, Rapsöl)
• 7 g vanilla extract (Taylor & Colledge, Bio Vanille Paste)
• 62.5 g gluten-free flour (Migros, Aha Mehlzubereitung glutenfrei)
• 2 g baking powder (Aldi, Bella Backpulver)
• Blue dye powder yielding 30 - 241 mg colorant per muffin (Günter Aroma AG, Hollinger Farbpulver Blau).

## Declaration of generative AI and AI-assisted technologies in the writing process

During the preparation of this work the authors used ChatGPT to shorten long text paragraphs to improve readability. After using this tool, the authors reviewed and edited the content as needed and take full responsibility for the content of the published article.

## CRediT authorship contribution statement

**Cyra Schmandt:** Investigation, Formal analysis, Writing – original draft. **Julia Trunz:** Investigation, Formal analysis, Writing – original draft. **Claudio Perret:** Conceptualization, Supervision, Writing – review & editing, Funding acquisition. **Anneke Hertig-Godeschalk:** Conceptualization, Writing – review & editing. **Zeno Stanga:** Writing – review & editing. **Jivko Stoyanov:** Conceptualization, Supervision, Writing – review & editing, Funding acquisition.

## Declaration of competing interest

The authors declare that they have no known competing financial interests or personal relationships that could have appeared to influence the work reported in this paper.

## Data Availability

Data will be made available on request.
